# “I’ve Redeemed Myself by Being a 1950s ‘Housewife’ ”: Parent–Grandparent Relationships in the Context of Lesbian Childbirth

**DOI:** 10.1177/0192513X14563798

**Published:** 2015-03

**Authors:** Petra Nordqvist

**Affiliations:** 1 University of Manchester, Manchester, UK

**Keywords:** fertility, intergenerational, LGBTQ issues, parent–child relations, same-sex relationships, sexuality

## Abstract

This article investigates the relationship between grandparents and lesbian daughters in the context of childbirth, looking specifically at the role that pregnancy plays in shaping kinship affinities. Gender, sex, and heterosexuality are fundamental to Euro-American kinship discourse and practice; lesbian couples’ parenthood through donor conception represents a significant departure from prevailing tropes of kinship. Thus, questions arise about how lesbians experience becoming and being parents, and about how their own parents may respond to becoming a genetic or nongenetic grandparent. This article draws on original data from interviews conducted in the United Kingdom with lesbians who became parents by donor conception, and grandparents with lesbian daughters in those situations where the older generation was not originally supportive of their daughters. It explores the negotiated meaning of pregnancy and how relationships with grandparents may be shaped by whether or not it is the daughter of the family who gave birth.

## Introduction

Euro-American kinship system is built on and structured by ideas of sex, gender, and (hetero)sexuality. In her seminal feminist analysis of a sex/gender system of kinship, Rubin (1997) argued that men and women are constructed in a political economy in which women are “trafficked” between men; she thereby made the point that kinship is *constituted* through binary constructions of gender. Taking this further, Yanagisako and Collier (1987) suggested both that men and women are culturally construed as different and that this difference is constructed as being based in nature; the fact that women bear children through pregnancy is a fundamental part of the perceived natural (or “biological”) differences. Strathern (1992) went on to show how sex, gender, and heterosexuality are all components of a system of kinship in which family relationships are culturally perceived as relationships “based in nature” through connections of blood or (more recently) genes. Schneider (1968/1980) neatly sums up the “logic” of kinship in the United States:

The members of the family are defined in terms of sexual intercourse as a reproductive act, stressing the sexual relationship between husband and wife and the biological identity between parent and child, and between siblings. (p. 51)

Lesbian couples who form families through the use of donor sperm contravene this kinship logic on several accounts. First, the same-sex relationship represents in itself a significant detour from the idea of the heterosexual couple being the basis for family life. Second, when a lesbian couple goes on to have children, the act of procreation does not produce the culturally expected mother and father, but instead it reproduces two mothers and a donor, in other words, a “cultural unknown.” Third, same-sex parenting upsets rules about genetic connections in the family as the grandparents on the side of the nongenetic mother have no genetic relationship with the child; *their* daughter has not been pregnant. Finally, being a nonbirth mother upsets ideas about womanhood because a perceived fundamental part of being a woman is to bear a child. In short, there is a compounding effect of “difference” in families created by lesbian mothers. Following Butler (2004), it could be argued that the traditional kinship logic produces grids for liveable lives that render lesbian motherhood quite unintelligible, even “unliveable.”

This conclusion, that lesbian motherhood is theoretically speaking “unliveable,” raises empirical questions. How do lesbian parents experience having children? What it is like for their own parents when they do so? How do conception and childbirth affect the relationships between the generations? How are children by donor conception with lesbian mothers received into the folds of the wider family? I explore these questions in this article, drawing on recent data from the United Kingdom (conducted with Professor Carol Smart). I note a salient dynamic that emerged between lesbian parents and their parents, namely, that grandparents’ reactions to learning about a daughter’s sexuality shaped the relational dynamics around childbirth, particularly when these initial reactions were negative. Not all lesbian mothers in the study lived with unsupportive family relations. However, I focus on those families where the grandparent generation had initially reacted badly to a daughter’s “coming out,” and where the daughter had become a kind of “relative stranger” in the family. In these families, the daughter’s lesbianism and her family-building process were felt to be both unfamiliar and incomprehensible to the parents (Nordqvist & Smart, 2014a). As I show in this article, within that relational context, being pregnant (or not) carried significant meaning for how relationships developed between “grandparents” and their lesbian daughters. I bracket the significance of the donor for now which has been explored elsewhere (Nordqvist, 2011, 2012) in favor of looking specifically at the significance of pregnancy for relational dynamics.

The phenomenon of same-sex couples having children is becoming increasingly common. Lesbian, gay, bisexual, and transgender relationships now enjoy improved social and legal recognition in the United Kingdom (Weeks, 2007) through the Civil Partnership Act 2004 and the recent passing of the Marriage (Same Sex Couples) Act 2013. Same-sex couples who choose to have children within the context of their relationship often use some combination of assisted reproductive technologies (sperm, egg, or embryo donation, and surrogacy) as a means of doing so (e.g., Dempsey, 2013; Lewin, 1993; Mamo, 2007; Nordqvist, 2011). The emergence of these new kinds of families is part of an overarching diversification and “opening up” of family life socially, culturally, and legally (Silva & Smart, 1999; Stacey, 1996; Weeks, Heaphy, & Donovan, 2001). This article contributes to the growing body of research shedding light on the everyday experiences of families living outside traditional, cultural kinship norms (see also, e.g., Almack, 2008; Hertz, 2009).

## Living Family Lives

To explore family relationships empirically, I distinguish between the cultural dimension of kinship discourse (outlined above) and the actual experience of *living* family lives. That is, I do not assume that kinship logic fully determines everyday relationships or that its principles of relating translate into practice in a straightforward, predictable way. Rather, I rely on the work of Finch and Mason (1993), who suggest that we understand life within family networks as emerging from ongoing negotiations. That is, Finch and Mason (2001) suggest that “doing” family connections is shaped by ways of relating, or what they call “relationalities.” In practice, Finch and Mason note, family relationships are imbued with subtle layers of meaning which take shape over time, through the ongoing (explicit and implicit) negotiations of family members. The framework of “doing family” offers a view on family relationships that highlights the need to take into account both the fact that families operate in subtle and intricate worlds and the fact that the members of families live in interlinked, often negotiated, lives (see also Nelson, 2006; Smart, 2007).

**Figure 1.**
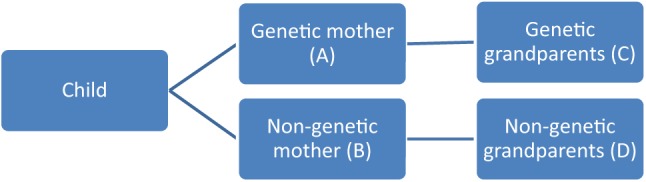
Child, parent, and grandparent relationships in lesbian mother families.

A series of “new” relationships emerge around a child in lesbian mother families and the ensuing complexity means that they require some further clarification. See Figure 1 for an illustration of these relationships.^1^ The relationship between the genetic mother (A), her parents (C), and the child falls close to normative notions of kinship because A carries the child. However, because A is in a lesbian relationship, relationships get more complicated, culturally speaking. In the place of a father, there is another (nongenetic) mother (B; and a genetically related donor, not marked in Figure 1) and so the family links that travel on the side of this mother are nongenetic. The nongenetic mother (B) has to come to terms with being a nongenetic parent and with not having carried the baby that she calls her own. Of course, this comes as no surprise: she and the partner decided that this is how they will have this child. B’s parents, the nongenetic grandparents (D) of A and B’s child, on the other hand, might have their own concerns. Had their adult child (B) been a man, there would have been an established cultural framework (genes) to account for their connection to their daughter-in-law (A; the genetic mother) and the child; a pregnancy would not have been a prerequisite to establish kinship links (Yanagisako & Collier, 1987). As it is, these parents (D) have become grandparents through their daughter (B) but she has neither been pregnant nor transmitted any genetic material to the baby (at least not in this study) and her partner is female. They are faced with the tasks of making sense of what it means for them to be “family-in-law” to the genetic mother, and deciding how to relate to the child, in the absence of there being any established social script for understanding this kinship affinity.

## Pregnancy and U.K. Law

Interpersonal negotiation operates within the context of law and policy which have powerful roles in shaping family relationships, perhaps especially within families that diverge from the norm. In the United Kingdom, legal motherhood is assigned through pregnancy (rather than, e.g., genetic relatedness), meaning that a woman who gives birth to a child (the birth mother) is legally that child’s mother. Until very recently, this meant that the process of pregnancy was enormously important for lesbian parents, because only the woman carrying the child was recognized as the legal “mother” whereas the nonbirth mother was legally invisible. This situation started to change in the early 2000s (prior to any regulation recognizing same-sex relationships in the United Kingdom), when the Adoption and Children Act 2002 made it possible for a gay or lesbian couple to file for adoption, thus securing legal parenthood for both parties. But inequalities persisted because unlike heterosexual couples conceiving using donor sperm (where it is assumed that the man is the legal father), the nonbirth parent in lesbian couples had to apply to adopt the child after birth. The situation shifted further after the Civil Partnership Act 2004, and more crucially after the Human Fertilisation and Embryology Act 2008, revising the regulating of provisions of reproductive donation in the United Kingdom. Through changes brought into law in 2009, lesbian couples who have accessed clinical treatment together, or are civil partners, can now both be recognized as the legal parents of the child even before birth. However, the category of “mother” remained unchanged and legally applies only to a woman who carries a baby. To recognize the role of a nonbirth mother, the category of “female parent” was added to the categories of “mother” and “father” (McCandless & Sheldon, 2014). Grandparents have no legal rights to grandchildren regardless of whether they are born into a heterosexual or same-sex relationship and regardless of whether or not they are genetically related.

## The Study

The data discussed in this article were collected as part of a broader project, which ran 2010 to 2013, to explore donor conception and nongenetic kinship within the context of wider family relationships, and to investigate the similarities and differences between heterosexual and lesbian family experiences.^2^ In this article, I focus only on the interviews conducted with lesbian parents and grandparents with lesbian daughters. Thirty-seven interviews (22 with parents and 15 with grandparents; 60 interviewees in total) form the basis of this article. Parent and grandparent interviewees were recruited from different families to avoid the risk of inadvertently conveying sensitive information during interviews and to ensure anonymity and confidentiality in writing. To further safeguard the research participants, names, places, and identifying details have been anonymized in the following accounts.

Recruitment and fieldwork took place in England and Wales in 2011. Most of the parent recruits came through the U.K. Donor Conception Network and local Lesbian Mums groups. The grandparents, who were a particularly hard-to-reach sample, were mainly recruited through parents of donor conceived children (who were not themselves taking part in the study) and through personal networks.

The lesbian parents and grandparents drew on experiences of sperm donation rather than egg or embryo donation (with the exception of one example in which a lesbian couple had one child through sperm donation and were expecting a sibling through embryo donation). The sample was split among those who relied on clinical conception (20), self-arranged conception (13), both clinical and self-arranged (2), and conception through an Internet company provider^3^ (2). All clinical conceptions took place in the United Kingdom, apart from the one case of embryo donation in which the couple had used a clinic abroad (in Eastern Europe). Aside from that one exception, all the birth mothers used their own rather than their partners’ eggs to conceive (and so they were also the genetic mothers). The majority of the 60 parent and grandparent interviewees were women; only six men (grandfathers) were among them. Fifty-one people (85%) in this group identified as White British and six identified as White European, American, or Australian; one identified as of mixed background and two as Chinese British. Over half of the interviewees (52%) in this group identified as atheist or agnostic; 37% identified as Christian (split in different faith groups) and 7% were Jewish. One person felt herself to be spiritual (two did not state their position on faith). Seventy-seven percent of the lesbian parents had gone on to higher education, which compared with the general population of women giving birth in Britain (Dex & Joshi, 2004) gives a broad indication that this group of parents was disproportionately middle class. The interviewees lived in both rural and urban locations in England and Wales, with particular concentration in Greater London and Manchester.

## Family Fallouts

In order to trace the pathways along which some lesbians negotiate becoming mothers and being pregnant (or not) in the context of relating to their own mothers and fathers, it is necessary to first take a step back and understand something about the history of the relationships in these families. Finch and Mason (1993) remind us of the importance of history and time in understanding family relationships, arguing that in order to understand the way in which family negotiations unfold in the present, we need to bring into view family biographies and the way in which relationships have unfolded along particular paths in the past.

“Coming out” as gay and lesbian to one’s family of origin remains a difficult and uncertain prospect (e.g., Nordqvist & Smart, 2014b). Many of our parent participants had found that the disclosure of their sexual orientation came as a shock and disappointment to their own mothers and fathers, leading relationships to strain and sometimes fracture.

Mum found [my coming out] very, very hard to come to terms with. She was blown away by the fact and my sister had to get her books on how to cope with your daughter being gay. (Dawn, with Linda, parent)

[My mum] didn’t react well [when I came out]. She said a psychologist would be a good idea. I didn’t pursue that (laughs). (Sasha, with Gemma, parent)

The dynamic of the story of pregnancy and motherhood that I unpack in this article was grounded in this kind of reaction from mothers and fathers, which could range from outright hostility, shock, and deep disappointment through to ambivalence, uncertainty, and general unsupportiveness. (As already noted, there were also lesbians in the broader study who had positive responses from their parents when coming out and grandparents who were fully supportive of their daughters’ families. These stories about family life tended to follow a different trajectory, and the meaning of pregnancy was also different.)

Many of our participants coped with family relationships where a previous “coming out” had caused family relationships to strain. Laura, who was married to Natalie, for example, recalled telling her mother and father about the beginning of that relationship:

I had a long-term boyfriend up until I was about twenty-six, twenty-seven, which [my mum and dad] were sure I was going to marry and, of course, then said, “No, I’ve met somebody else, it’s Natalie,” and it was the end of the world [to my mother]. (Laura, parent)

Laura’s mother had been distraught by the news of Laura starting a lesbian relationship, and she found it difficult to come to terms with that information that disrupted her expectation that Laura was following the expected heterosexual life trajectory. A similar negative reaction from parents was reported among women who had never given their parents reason to believe that they were heterosexual, this suggesting that the expectation on a heterosexual life trajectory remains deeply embedded in the family culture whether or not an individual gives evidence to support that pattern (e.g., Solebello & Elliott, 2011). Often, the mothers and fathers of lesbian daughters reacted as though assimilating a lesbian relationship into the family story was going to be both difficult and disagreeable. In Butler’s (2004) terms, it would appear that heterosexuality provided such a powerful grid for living a liveable life, that a life outside it appeared, at least initially, quite unintelligible to the parents of these adult children.

At the heart of these parents’ negative reactions were concerns about kinship and more specifically, lineage and grandchildren. Calhoun (2000) notes that gays and lesbians are perceived as “family outlaws,” a sort of “dead end” of the kinship line (Nordqvist & Smart, 2014b). In many cases, the issue of grandchildren was the particular sticking point causing a mother, especially, to react unfavorably to a daughter’s lesbianism. Nina recalled,

I can remember when I came out to my mum. It was one of the things that she really cried about, was, you know, “I won’t have children and she won’t have grandchildren from me.” And I think that was probably more of a loss for her than many other things associated with my coming out and being gay. (Nina, with Claudia, parent)

Although we did not interview two generations from the same family, we did hear the corresponding story of grandparents who found it very difficult to accept a daughter being gay. Veronica, a grandmother in the study, had been devastated by her daughter’s sexuality, in part because she thought it meant her daughter would never have children.

The fact that she was gay in the first place upset me terribly. [ . . . ] I found it terribly hard to accept that she wouldn’t have any children. I did talk to her about that and I don’t know if she felt pressured, or whether she wanted to or what, because I kept saying to her “how can you be like this and never have any children.” Because for me it’s such a big part of my life. And I felt very . . . very sad. And heartbroken. (Veronica, with Howard, grandparent)

Veronica kept her daughter’s “coming out” a secret to her own family and friends for a long time, and when she eventually told her own sister, the information was met with disgust and horror. In the end, Veronica sought out a support group for families of gays and lesbians to come to terms with her feelings. The perceived future without grandchildren was at the heart of her struggle.

Culturally, personally, and emotionally a new grandchild inhabits a special place in the relational network of kin, and especially so for those who have longed to one day become grandparents. A grandchild can of course change everyday life for grandparents because the transition can result in a new and extensive investment of time, care, and emotions (e.g., Fuller-Thomson & Minkler, 2001; Mason, May, & Clarke, 2007), but he or she might also be perceived as a carrier of hopes, dreams, and aspirations for the future. Nina and Veronica’s accounts give an indication about just how much can be invested in the idea of one’s children having their own children. Although grandparents are rarely entitled to influence their children’s reproductive decisions, they are nonetheless likely to be much affected by them (see also Nordqvist & Smart, 2014c).

As had Veronica, Nina’s mother, clearly took Nina’s announcement of being gay as a declaration that she would not ever have children. This assumption shows, perhaps, some lack of imagination in the beginning of the 2010s, but Nina’s mother (and others like her in the study) were of a generation growing up in the United Kingdom, when a homosexual son or daughter was at best tacitly accepted as part of the family, and at worst ousted and banished. Certainly, openly gay relationships, gay marriage, and childbirth within a gay relationship would not have been common or even possible during most of her lifetime and certainly not during her formative years (Nordqvist & Smart, 2014b; Weeks, 2007). It is hardly surprising therefore that some grandparents saw gay life as incompatible with parenthood.

Negative reactions from the older generation were deeply significant because they would set the scene for the ways in which relationships developed over time. Adult children lived their ongoing lives in same-sex relationships with the knowledge that, at best, their own parents coped with their choice of a female partner. In some families, the “coming out” caused the family relationship to rupture, with all parties having to cope with the strain this caused.

## Renegotiating Family Ruptures

These negative reactions constitute the relational context in which some of the women in same-sex relationships in our study went on to have children. Interestingly, we discovered that in some cases the decision to have children itself could lead to ruptured or strained relationships being rekindled. Indeed, a salient theme that emerged within our study was that the announcement of a pregnancy by a daughter could help older family members cope with her sexuality. The pregnancy became a vehicle to rekindle fractured family relationships because although the same-sex relationship was still an issue, the extended family was now able to come together in delight over a daughter being pregnant or, eventually, in a child being born into the family.

Veronica had worried that being a lesbian meant that her daughter would never have children. Howard, Veronica’s husband, noted that the birth of their daughter’s baby marked a new beginning for family dynamics:

When my wife first found out that her daughter was gay she took it very, very badly indeed. I think that really came to an end, the badness, really only came to an end when the baby was born. (Howard, with Veronica, grandparent)

The same dynamic of a pregnancy or birth improving relationships between generations was reflected in some of our interviews with lesbian parents who were well aware that their decision about who was going to carry the child was significant for their relationships with members of the wider family. Jessica was the genetic mother of her and Amy’s children. Jessica and Amy both were conscious that this fact shaped how Jessica’s mother received the family:

Jessica: I know my mum wouldn’t have felt as comfortable if it wasn’t me that was giving birth because of the whole. [ . . . ] Who knows what it would have been like, I just know my mum well enough to know that it would have mattered. [ . . . ]

Amy: That could have caused issues if I’d then gone on and had a second child and there could have been an upset. (Parents)

The couple had planned to give birth to one child each, but when Jessica fell pregnant with twins, they decided to not have more children. In response to Amy’s comment about a possible “upset,” Jessica said, “It’s not an issue because that’s not the way it turned out.”

Another example emerged in our interview with Meredith and Priscilla who had two children together: Priscilla had carried both boys. Priscilla’s mum disapproved of their lesbian relationship, and was initially also against the idea of Priscilla and Meredith having children together. At the time of the interview, however, this grandmother was not only delighted with the children but also lived locally and offered much practical support on a daily basis. However, both Priscilla and Meredith felt that this grandmother might have felt quite differently about her grandchildren had Meredith given birth:

If I’d phoned my Mum and said you know Meredith’s having a baby—we’re having a baby and Meredith’s the one that’s pregnant—I don’t think she would have thought of it as hers. (Priscilla, with Meredith, parent)

It was clear to both these mothers, that Priscilla’s giving birth had played an important role for Priscilla’s mother in terms of coming to terms with the lesbian relationship and recognizing the children as her grandchildren. Priscilla and her mother had reached an equilibrium in their relationship and enjoyed a close relationship, even though Priscilla felt her mother still did not accept her lesbianism:

My Mum doesn’t like the fact that I’m a lesbian but I think I’ve redeemed myself by being a fifties housewife (laughter). [ . . . ] I mean she’s happy that I’m happy but I think she would prefer if I was straight. (Priscilla, parent)

As this quote suggests, Priscilla and her mother had renegotiated the rupture in their relationship through Priscilla fulfilling a number of traditional notions of femininity, thus normalizing her life as a lesbian in the eyes of her own mother. Giving birth to the grandchildren was part of that process and so was leaving work to become a stay-at-home mother. In short, these accounts suggest that carrying a child was a way for a daughter to render her lesbian life intelligible to her own mother (and father) and slot back into a liveable life. None of lesbian couples in the study stated that their decision about who would carry the child was made to rebuild fractured relationships with the grandparents. Rather, this was a (very powerful) effect that the lesbian couples negotiated as part of that decision.

## Liveable Lives?

These accounts also suggest that for many grandparents it was crucially significant that it was *their* daughter who carried the child. This was clearly a problematic situation for the lesbian mothers, who were painfully aware that the goodwill of grandparents might be conditional. Couples knew that had the partner given birth instead, the grandparents may have reacted differently and they might not have recognized the child as a grandchild. Lesbian couples had to manage the reaction of grandparents, who, in some cases, failed to mirror the attitude of the lesbian couple who perceived themselves as equal parents to the children regardless of genetic connection. The conversation with Veronica and Howard suggests that their daughter would have had to manage this reaction had her partner, rather than she, had the baby:

Interviewer: If it had been [your daughter’s partner] who had actually had the baby, do you think that would have affected the way you feel about [your grandson]?

Veronica: Yes.

Howard: Oh yes. There wouldn’t have been any relationship at all.

Veronica: Well there would have been but it wouldn’t have been the same. No. Oh it would have made a hu–

Howard: It would have made huge difference.[ . . . ]

Veronica: Because it wouldn’t have been [my daughter’s] baby. (Grandparents)

It is clear from Veronica and Howard’s account that the daughter’s pregnancy is seen as fundamental for conferring a relationship between themselves and the grandchild. Howard suggests that there would simply not have been any relationship at all had his daughter not carried the child. Veronica, however, is a little bit more flexible in her approach. Nevertheless, she agrees that the daughter’s giving birth was hugely important. The meaning bestowed unto the pregnancy in this account appears to be one of “ownership” and “belonging”; due to the fact that the daughter being pregnant, it was *her* child, and thereby the child also *belonged* with Veronica and Howard.

Although we cannot tell for sure how Veronica and Howard might have related to a child carried by their daughter’s female partner, and whether their feelings might have softened over time, it was clear from other accounts in the study that it could take nongenetic grandparents some time to reach an understanding of the relationship. Tony and Anita were nongenetic grandparents who had different initial reactions from each other:

Tony : I mean I found it harder to accept than you [partner Anita] did I think really because I felt it was, I was sort of grandparent-in-law really because it wasn’t my daughter who was pregnant. Yeah but you know, after the first few months I sort of came to terms with that and I was very fond of [the boy] that’s for sure. It certainly took me longer than you I think didn’t it?

Anita : I adored him. He was my little boy. (Grandparents)

Focusing, in particular, on Tony’s account here, it appears that the kinship connection he had with the child felt confusing to him. By using the term “grandparent-in-law,” he appears to suggest that the child’s *real* grandparents are the genetic grandparents on the side of the genetic mother, whereas his relationship with the child is an “adopted” or “added” kinship rather than a “genuine” kind. It would seem that whereas his wife was able to sidestep cultural normative kinship blood ideologies, for Tony it was difficult to come to terms with exactly how he related to this child. His understanding of being related was so firmly defined by a matrix of heterosexuality, gender, and genes that connections outside that framework appeared unintelligible initially. This account also highlights that time is essential in many cases and that relationships shift and change as adverse reactions and feelings could soften.

Although the lesbian mothers were often able to renegotiate their relationship with their parents, this did not however necessarily mean that the grandparent generation fully accepted the lesbian relationship as a “proper” relationship or as a basis for a family. Some genetic grandparents who were quite supportive of their daughter and their genetic grandchild failed to recognize the place of the nonbirth mother in the family or to accept the family as a whole. One example of this emerged in our interview with Sheryl, whose parents had reacted very badly when she came out. She had formed a relationship with Penny and they had a child together through an informal donor relationship. Sheryl was the genetic mother. It was only when Sheryl gave birth that her parents started to warm to her again. Subsequently, the couple’s relationship broke down and Penny had gone on to have a second child on her own by the same donor. This second child was a little sister, both genetically speaking (through the donor) and socially speaking (through the nongenetic mother). The complexity clearly reflects the dynamics of lesbian family life and the sorts of relationships that can emerge in these families. Whereas Sheryl’s parents were taking a great interest in Sheryl’s birth child, they however failed to recognize any connection to the little sister.

[My parents] were just like, “Well that’s ridiculous [that they would be sisters] and it’s nothing to do with you.” You know, “You don’t want [your daughter] getting involved cause it’s going to mess her up and it’s going to affect her later on in life.” (Sheryl, parent)

It appears that whereas they accepted Sheryl’s daughter as “theirs” they never came to terms with the lesbian relationship, and disregarded any connection that Sheryl or the child might feel they had with the nongenetic mother Penny, the donor, or the baby sister.

Grandparents may look after the grandchildren for significant amounts of time, and be closely involved with the family, and yet harbor quiet discontent or show in subtle ways that they did not fully recognize the family relationships in place. Laura’s mother, for example, would look after their son every week and an issue arose around how she referred to the two mothers:

If I would go and pick him up from my Mum’s, then obviously [she would say to our son], “Oh, Mummy’s here or Mama’s here,” and we’d just sort of carry on. Natalie had commented, very recently actually, a few weeks ago [ . . . ] that she picked him up and my Mum said, “Natalie’s here.” Now, of course we never speak to [our son] or to anybody else and refer to Natalie as Natalie with him. You know, we’d say Mummy or Mum. [ . . . ] So I got on the phone straight away [to my mum]. (Laura, parent)

A flyaway comment from the genetic grandmother in the midst of everyday life indicated that she perceived her own daughter Laura, the child’s genetic mother, as the “real” mother whereas the partner by omission was not seen as a mother.

In other ways as well, grandparents’ unsupportive or ambivalent views could continue after childbirth, and parents and grandparents continually negotiated this part of their relationship. In many families relationships were deftly balanced in a fragile and precarious equilibrium that could quite easily be unsettled. This fragility left many of the lesbians feeling fearful of change in case it would lead to the loss of vital relationships. Sheryl, whose relationship with Penny had broken up, was a single mother at the time of the interview. Although she received much support from her parents, she worried that they would cut her and her daughter from the family if she met a new partner. Laura and Natalie were planning another child, but Laura struggled to conceive and so this time she was planning to try to get pregnant through IVF using one of Natalie’s eggs. She would carry the baby again but this time Natalie would be the genetic mother. Laura worried about how her mother might react, given her mother’s hesitancy to refer to Natalie as a mother:

Yeah, I suppose there’s a slight concern, again, would she [my mother] be difficult? You know, would she treat the child differently? Would she behave differently? Probably not, but obviously in my mind there’s the thought of “what if,” you know, “she did?” (Laura, parent)

## Strong and Vulnerable Kinship

Although our interviews only provide a snapshot in time, they did suggest that decisions about who would carry the child could have long-term significance and shape relationships as they developed over time. Vanessa and her partner had twins together carried by Vanessa. When the twins were 2 years old their nongenetic mother left. As Vanessa suggests, this brought a dramatic change in the relationships with her ex-partner’s (nongenetic) side of the family:

Members of her family have never so much as sent a postcard since the day [my partner] walked out. Nothing. Having been around a lot, having spent every summer with them from the first two years of their lives, I mean her mother was the person who came to stay for the week when they were born. Her entire family were up at the hospital with a boot full of baby things. They were all very much part of their life for the first 3 years but the day [my partner] left I never heard from anyone again. [ . . . ] But they were never very happy about her having children with a woman. They had accepted the children and they had adored the children but I felt . . . appalled at their part in it. Had I been a man they would have said, “They’re your children you stick at it” . . . but because I wasn’t, the relationships were so expendable it seemed. (Vanessa, parent)

It would appear that these grandparents found that the relationship with the children was only significant in so far as their daughter was in a relationship with the genetic mother. When the couple relationship fell apart, they seemed not to recognize any ongoing connection or commitment to the children. In this case any notion of being kin fell away, in complete contrast to cultural perceptions of the connection between a grandparent and a grandchild as a fixed relationship in the sense that it cannot be “dropped.” We cannot know the grandparents’ reasoning behind this, of course, but to Vanessa it was clear that it was linked to the fact that they never fully accepted the lesbian relationship and that the situation would have been different had the partner been a man and the children’s genetic father. It would appear that the combination of a lesbian relationship and nongenetically connected grandchildren resulted in relationships that were only in part recognizable as kin to the grandparents and that those relationships did not have the durability usually associated with kinship.

By way of contrast, because it was associated with being connected by genes and blood, a relationship conferred through pregnancy (even when that pregnancy occurred in the context of a lesbian relationship), might be perceived as a fixed and secure relationship even when put under strain. Louisa, a genetic grandmother in the study, found that her relationship with her granddaughter was only secure because it was her daughter who had given birth:

Louisa : [My daughter’s] partner made it very difficult for us to see [our granddaughter]. We saw her when she was 2 weeks old and we didn’t see her again till she was a year and a half. Because [my daughter’s] partner thought that her family was [the child’s] family, not [my daughter’s] family. So she made it very difficult. [ . . . ]

Interviewer : And if it hadn’t been [your daughter] that was the birth mother but the other woman who was the birth mother?

Louisa : We wouldn’t have been allowed to see her at all. (Grandparent)

Along the same lines, nongenetic relationships were experienced as fragile. This might be because the grandparents perceived them as such, as in Vanessa’s case, but it could also be because even though a set of grandparents loved and recognized a nongenetic child as their grandchild, there was, in some cases, little or no legal recognition of there being a kin relationship between the nongenetic mother and the child, meaning that they were hardly recognized as kin either. This was illustrated in our interviews with grandparents Betty and Richard, and Anita and Tony, who provide a useful reference point because unlike the other grandparents discussed in this article, they felt fully attached to their nongenetic grandchildren (as noted above, Tony took some time to come to terms with the situation). Betty and Richard had a 10-year-old nongenetic grandson who, in their own words, they felt was “theirs” from the moment he was born. They experienced themselves as family, but worried what might happen if the couple relationship broke down because of the lack of legal recognition afforded to the nongenetic mother. When their daughter secured legal parenthood through adoption, Betty and Richard were delighted and relieved.

Although this legal recognition that so comforted Betty and Richard is now afforded to nongenetic mothers as parents, the genetic relationship between the birth mother and the child is still given much weight in courts dealing with legal disputes between lesbian parents. This renders nongenetic connections truly fragile in the face of relationship breakdown (e.g., Millbank, 2008). Anita and Tony were facing this very situation because their daughter was in the middle of a difficult legal dispute since the daughter’s partner (the genetic mother) had left and taken the child with her. Anita, who was greatly attached to the boy, found herself in a desperate situation where she was prevented from having any contact with her grandchild. Parents as well as grandparents had to continually negotiate the ongoing lack of social, cultural, and legal recognition afforded to their kin relationships.

These accounts indicate that the pregnancy, and all that it represents, continues to structure family relationships as they go into the future. Depending on how connections are mapped in the family, they can make relationships seem protected and secure or lead to relationships breaking down or fading away. Mason (2008) suggests that genetic relationships are culturally perceived as “fixed,” and this can explain how a pregnancy could continue to protect relationships because it was understood as something that made relationships both strong and nonelective. In contrast, nongenetic relationships were in some families more vulnerable in the face of change. These perceptions are echoed in the legal understanding of motherhood: nongenetic parents and grandparents have reason to worry because the law does not recognize “social” relationships in the same way as “genetic” ones (Millbank, 2008; Smart, 2010).

## Concluding Thoughts

There is no social script for how to “do” kinship in a family where normative ideas of gender, sex, heterosexuality, and genetic relatedness do not apply. Based on the data presented here, lesbian family life raises questions in some family networks because it transgresses the traditional ways in which families relate; introducing a same-sex intimacy into the extended family can be the point at which close relationships between generations rupture. Not all families react negatively to a same-sex relationship, of course, but it is sociologically important to recognize that for many lesbians the journey to family life entails negotiating unsupportive or ambivalent reactions from their own parents.

It is within the context of unsettled relationships that pregnancy takes on the particular meaning discussed here. In these families, the announcement of a pregnancy spurred relationships with prospective grandparents to rekindle. The pregnancy operated in these families as a “peg” onto which to hang confusing kinship relationships, making those relationships intelligible to the wider family. In other words, pregnancy forged people’s thinking about relationships and framed relations as those of kin. Through the pregnancy, families renegotiated their kinship connection, meaning that relationships took a new, more positive, turn.

Becoming pregnant and giving birth thus acted as powerful processes of normalizing lesbian life and making the unfamiliar familiar. Lewin (1993) suggests that having children normalizes lesbian life. I would add that being pregnant plays a vital role in that process of normalization, especially for the genetic mother who is able to renegotiate relationships with her parents. I have argued that the event of a pregnancy allowed families of origin to overlay a daughter’s lesbian life with conventional kinship properties, making it intelligible. It does not necessarily mean, however, that they fully recognized the lesbian mother family as a “proper” family unit.

There is now an abundant literature on the meaning of genetic and social kinship in the context of assisted reproduction and donor conception (e.g., Blake, Casey, Readings, Jadva, & Golombok, 2010; Blyth & Frith, 2009; Edwards, 2000; Thompson, 2005). By way of contrast, the meaning of pregnancy is less well explored, especially when it occurs in the context of lesbian donor conception. The data presented in this article shed light on the importance of exploring that meaning. Pregnancy carries conceptual meaning in terms of conferring a perceived relationship in the body between generations and as such its meaning is associated with genes and blood. But the data indicate that it also carries relational meaning; it is experienced and used as a sign of femininity and womanhood, a key component of constructing a conventional female life trajectory. Pregnancy is further understood to confer ownership and to be the pathway through which children come to belong in families. Moreover, being pregnant is a practice and process that appears to connect mothers and daughters because it is a shared experience that bears witness of a shared, desired life (a life with children). A grandmother can feel closer to a daughter because they have both experienced pregnancy and childbirth, and so links are forged in memories, biographies, the everyday, and relationships. This suggests that pregnancy plays an important part in how we “do” kinship and how kinship is in fact constituted through everyday actions.

In sum, I argue that pregnancy carries powerful multidimensional conceptual and relational meaning that links in with biographies, memories, cultures, and gender in families.

My findings add to debates about change and continuity in family life. Although there have been immense structural changes to intimate relationships, enabling lesbians to form families who are socially and legally supported, conventional tropes of kinship remain powerful in everyday understanding of family connections. It appears that in some families, new (lesbian) relationalities become intelligible insofar as they fit with “old” kinship thinking. In such families, new properties of kinship (a lesbian couple having a child together) make sense to others only insofar as they adhere to gendered and genetic understandings of being related (a woman becoming a mother by virtue of being pregnant). This kinship thinking recycles notions of “fixed” rather than flexible connection (Mason, 2008) and tells of the significance of traditional tropes of connectedness, ownership, and belonging in personal life.

## Notes

1. I use the terms “genetic” or “nongenetic” parent/grandparent to indicate how relationships between the generations are structured through pregnancy in a particular family. I am, however, mindful of how this may contradict a more negotiated meaning of pregnancy, but I see the term as a less-than-perfect but acceptable linguistic shorthand to structurally describe family relationships.

2. For a full account of the larger study, see Nordqvist and Smart (2014a).

3. Internet company providers of donor sperm were available for a few years in the United Kingdom during the 2000s. Since 2007, they can no longer operate because a company needs a license from the Human Fertilisation and Embryology Authority to do so and guidelines only permit companies to use frozen (not fresh) sperm.
